# *In Situ* Localization and Rhythmic Expression of Ghrelin and *ghs-r1* Ghrelin Receptor in the Brain and Gastrointestinal Tract of Goldfish (*Carassius auratus*)

**DOI:** 10.1371/journal.pone.0141043

**Published:** 2015-10-27

**Authors:** Aída Sánchez-Bretaño, Ayelén M. Blanco, Suraj Unniappan, Olivier Kah, Marie-M. Gueguen, Juan I. Bertucci, Ángel L. Alonso-Gómez, Ana I. Valenciano, Esther Isorna, María J. Delgado

**Affiliations:** 1 Departamento de Fisiología (Fisiología Animal II), Facultad de Biología, Universidad Complutense, Madrid, Spain; 2 Laboratory of Integrative Neuroendocrinology, Department of Veterinary Biomedical Sciences, Western College of Veterinary Medicine, University of Saskatchewan, Saskatoon, Canada; 3 Inserm (Research Institute for Health, Environment and Occupation, IRSET), SFR Biosit Université de Rennes 1, Rennes, France; 4 Instituto de Investigaciones Biotecnológicas-Instituto Tecnológico Chascomús, Buenos Aires, Argentina; Centre of Marine Sciences & University of Algarve, PORTUGAL

## Abstract

Ghrelin is a gut-brain peptide hormone, which binds to the growth hormone secretagogue receptor (GHS-R) to regulate a wide variety of biological processes in fish. Despite these prominent physiological roles, no studies have reported the anatomical distribution of *preproghrelin* transcripts using *in situ* hybridization in a non-mammalian vertebrate, and its mapping within the different encephalic areas remains unknown. Similarly, no information is available on the possible 24-h variations in the expression of *preproghrelin* and its receptor in any vertebrate species. The first aim of this study was to investigate the anatomical distribution of ghrelin and GHS-R1a ghrelin receptor subtype in brain and gastrointestinal tract of goldfish (*Carassius auratus*) using immunohistochemistry and *in situ* hybridization. Our second aim was to characterize possible daily variations of *preproghrelin* and *ghs-r1* mRNA expression in central and peripheral tissues using real-time reverse transcription-quantitative PCR. Results show ghrelin expression and immunoreactivity in the gastrointestinal tract, with the most abundant signal observed in the mucosal epithelium. These are in agreement with previous findings on mucosal cells as the primary synthesizing site of ghrelin in goldfish. Ghrelin receptor was observed mainly in the hypothalamus with low expression in telencephalon, pineal and cerebellum, and in the same gastrointestinal areas as ghrelin. Daily rhythms in mRNA expression were found for *preproghrelin* and *ghs-r1* in hypothalamus and pituitary with the acrophase occurring at nighttime. *Preproghrelin*, but not *ghs-r1a*, displayed a similar daily expression rhythm in the gastrointestinal tract with an amplitude 3-fold higher than the rest of tissues. Together, these results described for the first time in fish the mapping of *preproghrelin* and ghrelin receptor *ghs-r1a* in brain and gastrointestinal tract of goldfish, and provide the first evidence for a daily regulation of both genes expression in such locations, suggesting a possible connection between the ghrelinergic and circadian systems in teleosts.

## Introduction

Ghrelin, a peptide hormone mainly synthesized by the gut, was originally purified in 1999 by Kojima and colleagues [1]. The main site for the synthesis of ghrelin in all the vertebrates so far studied is the stomach or its equivalent [2], although gene expression of ghrelin by PCR shows a widespread tissue distribution, with low expression levels in peripheral tissues (apart from stomach) and brain in both mammals [3,4] and fish [5–7]. Imaging techniques have reported the presence of ghrelin gene and peptide in the brain [8] and gastrointestinal tract [9–12] of mammals. Similarly, ghrelin peptide has been localized by immunohistochemistry in the hypothalamus of goldfish [13] and in the gastrointestinal tract of goldfish (*Carassius auratus*) [14], sea bass (*Dicentrarchus labrax*) [14], zebrafish (*Danio rerio*) [15], and rainbow trout (*Oncorhynchus mykiss*) [16]. However, no studies to date have reported the anatomical distribution of the *preproghrelin* transcripts using *in situ* hybridization in a non-mammalian vertebrate, and its mapping within the different encephalic areas remains unknown.

Ghrelin is suggested to have a key role in energy balance regulation by promoting food intake, carbohydrate utilization and adiposity [17,18], and other physiological processes [19,20]. A unique aspect of this peptide is the presence of a posttranslational acyl modification catalysed by a recently discovered member of the membrane-bound O-acyltransferase family, named ghrelin O-acyl transferase [21,22]. This modification is essential for most of the bioactivity of the peptide, enabling the binding to its receptor, the G protein-coupled growth hormone secretagogue receptor (GHS-R). In contrast to mammals and other tetrapods with only one GHS-R gene, ancestral teleost underwent a genome duplication, and two paralog genes (GHS-R1 and GHS-R2) have been identified in otophysi teleosts [23]. Particularly, goldfish has experienced a tetraploidization, and four subtypes of GHS-Rs have been described and characterized in this teleost, named GHS-R1a1, GHS-R1a2, GHS-R2a1, and GHS-R2a2 [23]. In addition, each one of these receptor subtypes presents a type ‘b’ isoform obtained by alternative mRNA splicing. Among all of these different GHS-R, the GHS-R1a has been widely studied in vertebrates in terms of structure, tissue abundance, mechanism of action, dynamics and regulation, and seems to be involved in many of the physiological actions of ghrelin [3,24,25]. Nevertheless, little information is available about its anatomical location and distribution. In this sense, GHS-R1a has been localized in the mammalian brain [26–28], but no neuroanatomic mapping using imaging techniques has been performed in the brain of non-mammalian vertebrates. Among fishes, the distribution of ghrelin receptor in the gastrointestinal tract has been described only in zebrafish [15].

Most behaviour and physiology of living organisms follow daily rhythms due to the presence of endogenous clocks that synchronize biological processes to the 24-h light/dark cycle, enabling them to anticipate periodic changes in the environment. A growing interest in the relationships between energy balance and the circadian system has occurred in the last years. In fact, daily oscillations have been found in fish for many hormones involved in food intake regulation and metabolism, including neuropeptide Y (NPY) [29] and leptin [30]. In relation with the ghrelinergic system, only one study carried out in humans described the 24-h secretion profile of ghrelin, showing a circadian oscillation of this peptide with higher levels of circulating ghrelin during the night [31]. However, no information is available about the possible 24-h variations in the expression of this hormone and its receptor in vertebrates.

Goldfish is a member of the teleostean Cyprinidae family and has been widely used for studying the hormonal regulation of feeding in fish [32,33]. The biological activities of ghrelin have been previously examined in this teleost, [34–37] but, despite the growing interest and importance of the ghrelinergic system, no studies have reported a brain mapping of the elements composing this system in fish, and nothing is known about its possible rhythmic expression. Therefore, the aim of the present study was first, to investigate the anatomical distribution of ghrelin and the ghrelin receptor subtype GHS-R1a in the brain and gastrointestinal tract of goldfish by using immunohistochemistry and *in situ* hybridization techniques. We also characterized the daily profile of *preproghrelin* and *ghs-r1* mRNA expression in both central and peripheral tissues using RT-qPCR.

## Material and Methods

### Ethics statement

All procedures carried out in France were conducted in accordance with the guidelines of Ethical Committee at our institutions (University of Rennes 1, CNRS and INSERM) and in accordance with European Union regulations concerning the protection of experimental animals (Directive 86/609/EEC). The protocols were approved by the Ethical Committee CREEA (Comité Rennais d'Ethique en matière d'Expérimentation Animale) and performed under the supervision of authorized investigators (Permit number: EEA B-35-040). The study conducted in Canada strictly followed the regulations of the Canadian Council for Animal Care and were approved by the University of Saskatchewan Animal Research Ethics Board (Protocol # 2012–0082). The euthanasia was performed by deep anesthesia and all efforts were made to minimize suffering.

### Animals, experimental designs and sampling

For the anatomical studies, goldfish (2.0 ± 0.5 g) obtained from a local supplier (Rennes, France) were maintained in 60 l aquaria with filtered and aerated fresh water (22 ± 1°C) under a 12 h light: 12 h darkness (12L:12D) photoperiod (lights on at 9 AM). Fish were daily fed at *zeitgeber* time 2 (ZT-2, or 11 AM, as ZT-0 corresponds to lights-on) with food pellets (1% body weight; Novo GranoMix, JBL, GmbH & Co., Neuhofen, Germany). Goldfish were fasted for 48 h, and at ZT-2 of the experimental day were anesthetized with phenoxyethanol 1 ml/l (ICN Biomedicals Inc., Irvine, CA, USA) and sacrificed. Then, fish were immersed overnight in 4% paraformaldehyde diluted in 0.1 M sodium phosphate buffer with saline (PBS, pH 7.4). The following day, the brain and the gastrointestinal tract (esophagus, intestinal bulb, j-loop and anterior intestine) were removed and post-fixed 3 h in the same solution. Then, the samples were cryoprotected with 30% sucrose (MP Biomedical, LLC, Illkirch, France) overnight, included in the frozen section medium Richard-Allan Scientific^TM^ Neg-50 (Thermo Shandon Scientific, Cheshir, UK) and stored at -80°C.

For the study of daily changes in gene expression, goldfish (22 ± 8 g) obtained from a commercial supplier (Aquatic Imports, Calgary, Alberta, Canada) were maintained in 200 L aquaria with filtered and aerated fresh water (21 ± 2°C). Fish were maintained under a 12L:12D photoperiod (lights on at 7 AM) and daily fed at ZT-4 with a commercial pellet diet (1% body weight; Martin Profishent, Ontario, Canada). The day of the experiment, fish were sacrificed in 4 h intervals (6 fish/sampling time) throughout a complete 24-h cycle beginning at ZT-0. Fish were randomly collected from two different tanks (21 fish/tank). Food was offered as scheduled the day of the experiment. Once sacrificed, samples of forebrain (including telencephalon and diencephalon without hypothalamus), hypothalamus, hindbrain (including mesencephalon and rombencephalon), pituitary and gastrointestinal tract (including esophagus, intestinal bulb, j-loop and anterior intestine) were collected and immediately frozen in liquid nitrogen. Sampling during darkness was conducted under dim red lighting.

### Molecular cloning of *preproghrelin* and *ghs-r1a* genes of goldfish and riboprobe synthesis

For the molecular cloning of *preproghrelin* and *ghs-r1a* genes, specific primers (Table 1) were designed based on the goldfish sequences (GenBank accession numbers AF454389.1 for *preproghrelin*, AB504275.1 for *ghs-r1a1*, and AB504276.1 for *ghs-r1a2*) and were purchased from Sigma (Sigma-Aldrich, Steinheim, Germany). To clone *preproghrelin* gene (366 bp), the reaction mixture contained cDNA from goldfish gastrointestinal tract, GoTaq® Green MasterMix (Promega, Madison, WI, USA), and forward and reverse primers (3 nM each). To clone *ghs-r1a* gene (979 bp), the reaction mixture contained cDNA from goldfish gastrointestinal tract, Taq DNA Polymerase with its buffer (0.00125 U; Invitrogen), dNTPs (10 mM each), MgCl_2_ (6 mM), and forward and reverse primers (3 nM each). These primers were able to distinguish between the *ghs-r1a* and *ghs-r1b* splicing variants, but unable to distinctly identify *ghs-r1a1* and *ghs-r1a2* subtypes. PCRs were performed in a total volume of 25 μl. PCR conditions were set at 95°C for 3 min followed by 40 cycles of 95°C for 10 sec, 60°C for 30 sec and 72°C for 1 min, and a final extension step of 72°C for 10 min. The amplified products were run on an agarose gel and purified using PCR clean-up gel extraction (MACHEREY-NAGEL GmbH & Co., Düren, Germany) for *preproghrelin* gene and GenElute gel extraction kit (Sigma, Steinheim, Germany) for *ghs-r1a* gene. Purified *preproghrelin* and *ghs-r1a* PCR products were ligated into pCR®II- TOPO® (Invitrogen, Carlsbad, CA, USA) or pCR™4-TOPO® (Invitrogen, Carlsbad, CA, USA) vectors, respectively, and employed to transform *Escherichia coli* One Shot TOP10 cells (Invitrogen, Carlsbad, CA, USA) or JM109 cells (Promega, Madison, WI, USA), respectively. Positive clones were collected and plasmid DNA extraction was performed by using a routine miniprep protocol. Plasmids with the insert were linearized with *SpeI* and *NotI*. Antisense and sense mRNA probes were obtained with DIG RNA labeling MIX (Roche Diagnostic, Mannheim, Germany) by *in vitro* transcription with T7 and SP6 RNA polymerases (Promega, Madison, WI, USA) for *preproghrelin* gene, and with T7 and T3 RNA polymerases (Promega, Madison, WI, USA) for *ghs-r1a* gene probes. The specificity of the probes was confirmed with parallel series of slides hybridized with the correspondent sense RNA probes.

**Table 1:** Accession numbers of genes and primer sequences used in this study.

Target gene	GenBank Accession number	Primer sequences 5’ → 3’		Product (bp)	Application
*preproghrelin*	AF454389.1	F	GCAGCCATTCAGAGTGTTGT	366	Riboprobe synthesis
		R	CAGAATTCAAGTGGCGAATC		
		F	ATTCAGAGTGTTGTCGTA	103	RT-qPCR
		R	AGGAAAGAGCACATAAGA		
*ghs-r1a*	AB504275 (*ghs-r1a1*) AB504276 (*ghs-r1a2*)	F	GAACCGGTCCAACTGTTCCT	979	Riboprobe synthesis
		R	AAGTTTGCAAGCTGCCATCC		
*ghs-r1*	AB504275 (*ghs-r1a1*) AB504276 (*ghs-r1a2*)	F	ATTCGAGCACCCGGTCAACA	207	RT-qPCR
		R	TCCAGGGGCATGCAGAGAAA		
*β-actin*	AB039726.2	F	CTACTGGTATTGTGATGGACT	579	RT-qPCR
		R	TCCAGACAGAGTATTTGCGCT		
*elongation factor 1α*	AB056104	F	CCCTGGCCACAGAGATTTCA	101	RT-qPCR
		R	CAGCCTCGAACTCACCAACA		
*18s*	FJ710820.1	F	GGATGCCCTTAACTGGGTGT	206	RT-qPCR
		R	CTAGCGGCGCAATACGAATG		

F: forward, R: reverse

### Localization of *preproghrelin* and *ghs-r1a* by *in situ* hybridization (ISH)

The presence and anatomical distribution of *preproghrelin* and *ghs-r1a* transcripts in the brain and gastrointestinal tract of goldfish were studied by *in situ* hybridization. For this purpose, samples obtained as described earlier were embedded in TissueTek and sectioned at 8-μm thickness using a cryostat. Transverse sections were mounted onto superfrost slides (Thermo scientific, Braunschweig, Germany). The protocol for ISH was performed as previously described [38] with minor modifications. In brief, cryostat sections were washed in PBS two times during 10 min before post-fixation in *Antigenfix* (DiaPath, Martinengo, Italy) for 20 min. After washing in PBS, sections were treated for 5 min at 37°C with proteinase K (2 μg/ml, Sigma, Steinheim, Germany) diluted in PBS, and fixed in 4% paraformaldehyde for 15 min. Sections were rinsed twice in 2X standard saline citrate (SSC). Hybridization was performed at 65°C overnight in a humidified chamber using 100 μl hybridization buffer (50% deionized formamide; 2x SSC; 5x Denhardt's solution; 50 μg/ml of yeast tRNA; 4 mM EDTA; 2.5% dextran sulfate) containing the DIG-labeled probe (3 μg/ml). After hybridization, slides were washed in 2x SSC at 65°C (2x30 min), 2x SSC/50% formamide at 65°C (2x30 min), 0.2x SSC (1x15 min) and 0.1x SSC (1x15 min) at room temperature. Slides were next washed in 100 mM Tris-HCl (pH 7.5) containing 150 mM NaCl for 10 min, then washed in the same buffer containing 0.1% Triton and 0.5% of skimmed milk powder (2x30 min), and incubated overnight at room temperature with anti-digoxigenin alkaline phosphatase Fab fragments (1:2,000; Roche Pharma, Mannheim, Germany). The next day, slides were incubated for 4.5 h with an HNPP (2-hydroxy-3-naphtoic acid -2′-phenylanilide phosphate)/FastRED detection kit (Roche Pharma, Mannheim, Germany), according to the manufacturer's instructions. Finally, Vechashield mounting medium containing 4′,6-diamidino-2-phenylindole (DAPI; Vector Laboratories, Burlingame, CA, USA) was applied and coverslips were placed. Slides were observed with an epifluorescence microscope (Olympus Provis, equipped with a DP71 digital camera). Images were processed with either the Olympus Analysis or Zeiss Cell software. Micrographs were generated in the “TIFF” format and adjusted linearly for light and contrast before being assembled on plates using Photoshop CS6.

### Localization of ghrelin by immunohistochemistry (IHC)

Immunohistochemical staining of goldfish brain and gastrointestinal tract samples was used to study the anatomical distribution of ghrelin. The immunohistochemistry study was carried out as previously described [39]. Briefly, the above described cryostat sections were washed twice in 0.1M PBS and incubated twice in PBS containing 0.2% Triton and 0.5% of skimmed milk powder (45 min at room temperature). After overnight incubation with primary monoclonal antibody (mouse anti human ghrelin 1:200; ab57222, Abcam, Cambridge, MA, USA), previously used in goldfish by Kerbel and Unniappan [13], sections were washed three times in 0.2% Triton PBS and subsequently incubated with rabbit antimouse Alexa Fluor 488 (1:400; Invitrogen Molecular Probes, Eugene, OR, USA) for 2 hours at room temperature. A separate set of negative control slides were only treated with the secondary antibody (S1 Fig). After washing in PBS, slides were coverslipped with Vectashield containing DAPI and observed with an epifluorescence microscope (Olympus Provis, equipped with a DP71 digital camera). Imaging processing was conducted as described previously for ISH.

### Analysis of daily *preproghrelin* and *ghs-r1* mRNA expression by RT-qPCR

The possible 24-h rhythmic expression of *preproghrelin* and *ghs-r1* in the brain and gastrointestinal tract of goldfish was studied using Real-time or Reverse Transcription-quantitative PCRs (RT-qPCR). Total RNA from forebrain, hypothalamus, hindbrain, pituitary and gastrointestinal tract was isolated using TRIzol RNA isolation reagent (Invitrogen, Carlsbad, CA, USA). RNA purity was validated by optical density absorption ratio (260/280 nm) using a NanoDrop 2000c (Thermo, Vantaa, Finland). Then, an aliquot of 1 μg of total RNA was reverse transcribed into cDNA in a 20 μl reaction volume using iScript cDNA synthesis kit (BioRad, Hercules, USA) according to the manufacturer’s instructions. The RT reactions condition consisted in 25°C for 5 min, an extension of 30 min at 42°C and a denaturalization step at 85°C for 5 min, and were carried out in a T100 Thermal Cycler (BioRad, Hercules, USA).

RT-qPCRs were performed using iQ SYBR Green Supermix (BioRad, Hercules, USA). The specific primer sequences used for target genes *preproghrelin* and *ghs-r1*, and reference genes *β-actin* (accession number AB039726.2), *elongation factor 1α* (EF1α; accession number AB056104) and *18s* (accession number FJ710820.1) were ordered to IDT (Ontario, Canada) and are shown in Table 1. Primers used for quantifying ghrelin receptor were designed on the exon 1 (common for *ghs-r1a* and *ghs-r1b* splicing variants) and in a region conserved between the *ghs-r1a1* and *ghs-r1a2* sequences, so PCR products correspond to the sum of all *ghs-r1* mRNA isoforms mentioned. Genes were amplified in duplicated qPCR runs using a 96-well plate loaded with 1 μL of cDNA and 500 nM of each forward and reverse primer in a final volume of 20 μL. Each PCR run included a standard curve for the corresponding gene made of two replicates of three serial dilution points and water controls to ensure that the reagents were not contaminated. RT-qPCR cycling conditions consisted of a ramp of 95°C for 5 min, 35 cycles of 95°C for 30 sec, 56.6°C/60°C (*preproghrelin* and *ghs-r1*, respectively) for 30 sec and 73°C for 30 sec, and a final step of 95°C for 10 min. A melting curve was systematically monitored (temperature gradient at 0.5°C/5 sec from 65 to 95°C) at the end of each run to confirm the specificity of the amplification reaction. In addition, PCR products were electrophoresed on a 1% agarose gel, and single bands for each gene were purified using GenElute™ Gel Extraction Kit (Sigma-Aldrich, Madrid, Spain) and sequenced (Secugen, Madrid, Spain). The efficiency of the amplification for all studied genes was around 100%. All runs were performed using a CFX Connect Real-time System (BioRad, Hercules, USA). The 2-ΔΔCt method [40] was used to determine the relative mRNA expression, assigning the relative value of ‘1’ to the sampling time with the lowest expression values.

### Statistics

Analysis of mRNA relative abundance among time points was conducted using one-way ANOVA followed by post-hoc Student-Newman-Keuls multiple comparison test. All analyses were carried out using SigmaStat 12.0 statistics package. In addition, to evaluate rhythmicity of gene expression, cosinor analysis was performed by fitting periodic sinusoidal functions to the expression values for the genes across the seven time points. The formula used was f(t) = M+Acos(tπ/12−Π), where f(t) is the gene expression level in a given time, the mesor (M) is the mean value, A is the sinusoidal amplitude of oscillation, t is time in hours and Π is the acrophase (time of peak expression). Non-linear regression allows the estimation of M, A, and Π, and their standard error (SE) [41]. Significance of cosinor analysis was tested using the zero-amplitude test, which indicates if the sinusoidal amplitude differs from 0 with a given probability [42]. The time series data were considered to display a significant 24-h rhythm when p<0.05 by ANOVA, and p<0.005 by the zero-amplitude test with cosinor analysis.

## Results

### Brain and gastrointestinal distribution of *preproghrelin*, ghrelin and *ghs-r1a* in goldfish

The *preproghrelin* and *ghs-r1a* mRNAs were observed surrounding the nucleus of the cells (A, B, D, E in S1 Fig), while the sense riboprobes yielded no signal (C, F in S1 Fig), supporting the specificity of the obtained signal under the conditions employed. Only some blood cells show unspecific labeling in both sense and antisense riboprobes (C, F in S1 Fig). For the immunohistochemical analysis, specific signal of the peptide is observed at the cytoplasm level in the gastrointestinal tract cells (G in S1 Fig) and no staining was observed in the control sections stained only with the secondary antibody (H in S1 Fig).

**Fig 1:**
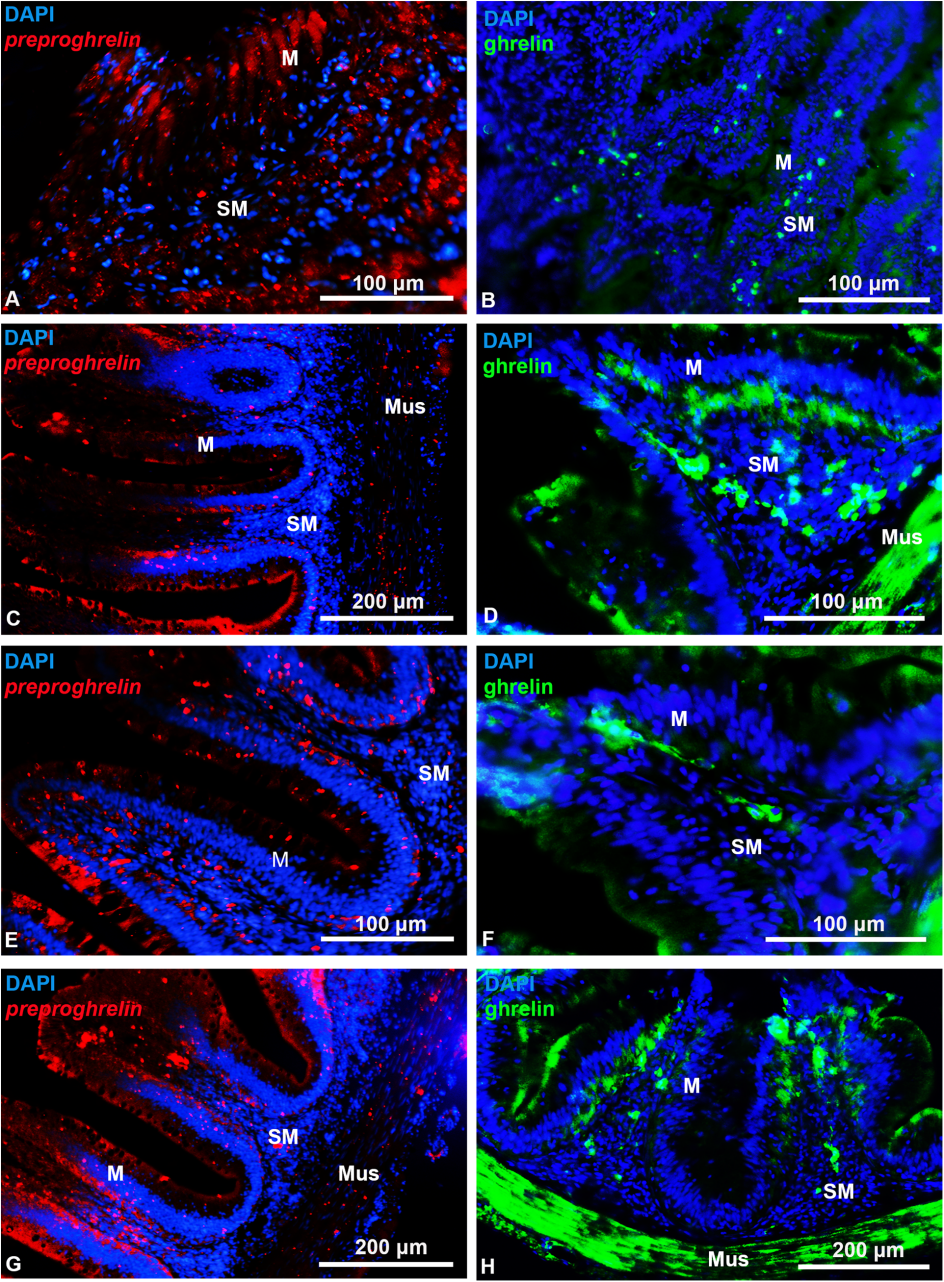
Transversal representative sections of goldfish gastrointestinal tract showing *preproghrelin* positive cells identified by *in situ* hybridization (left panel) and ghrelin immunoreactive cells detected by immunohistochemistry (right panel). (A, B) Esophagus. (C, D) Intestinal bulb. (E, F) j-loop. (G, H) Anterior intestine. M, mucose; Mus, muscular layer; SM, submucose. Scale bars are indicated in each image.

*Preproghrelin mRNA* expression and ghrelin were found by ISH and IHC in the mucosal and submucosal layers of all the sections analyzed from the esophagus to the anterior intestine (Fig 1). *Preproghrelin* transcripts were widely expressed along the esophagus (Fig 1A), while in the intestinal bulb, j-loop and anterior intestine, signal of *preproghrelin* probes was mainly located in the most basal cells of the mucosal epithelium and in the submucosal layer, with lower expression in the muscular layer (Fig 1C, 1E and 1G). Ghrelin-like immunoreactivity (ir) was higher in cells of the submucosal layer in the esophagus, intestinal bulb and j-loop, while in the anterior intestine, it was predominant in cells of the mucosal layer although still detected in the submucosa (Fig 1B, 1D, 1F and 1H). Ghrelin-like ir was also observed in the muscular layer of the gastrointestinal tract (Fig 1D and 1H). *Preproghrelin mRNA* expression and ghrelin were undetected in goldfish brain neither by ISH nor by IHC.

**Fig 2:**
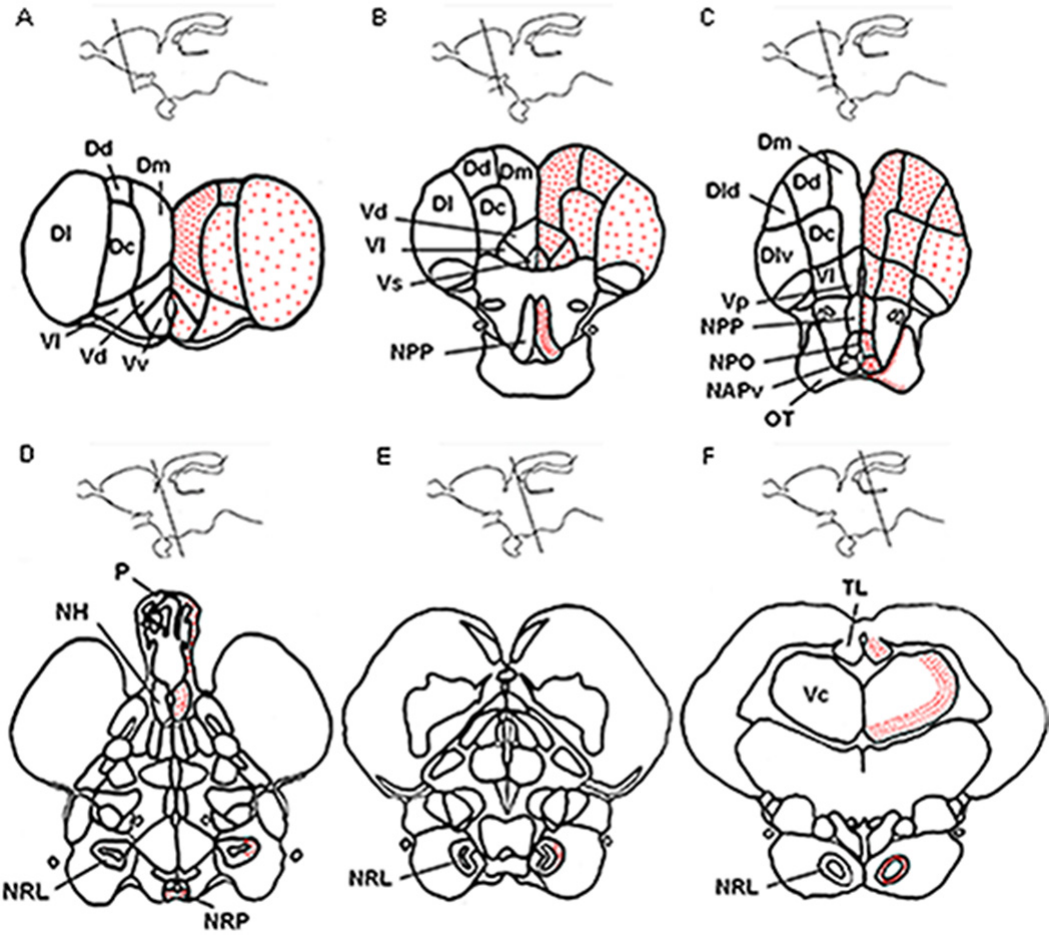
Schematic representation of *ghs-r1a* expressing cells in antero-posterior (A to F) transversal sections of goldfish brain [*Carassius auratus* Forebrain Atlas [43]]. The brain areas shown in the transversal sections are indicated with a schematic representation in the upper part of each image. Intensity of signal is represented by density of red dots. Dc, central portion of the dorsal telencephalon; Dd, dorsal portion of the dorsal telencephalon; Dl, lateral portion of the dorsal telencephalon; Dld, dorsal part of the lateral portion of the dorsal telencephalon; Dlv ventral part of the lateral portion of the dorsal telencephalon; Dm, medial portion of the dorsal telencephalon; NAPv, anterior periventricular nucleus; NH habenular nucleus; NPO, preoptic nucleus; NPP, periventricular preoptic nucleus; NRL, lateral recess nucleus; NRP, posterior recess nucleus; OT, optic tract; P, pineal; TL, torus longitudinalis; Vc, valvula of the cerebellum; Vd, dorsal portion of the ventral telencephalon; Vl, lateral portion of the ventral telencephalon; Vp, postcommissural portion of the ventral telencephalon; Vs, supracommisural portion of the ventral telencephalon; Vv, ventral portion of the ventral telencephalon.

**Fig 3:**
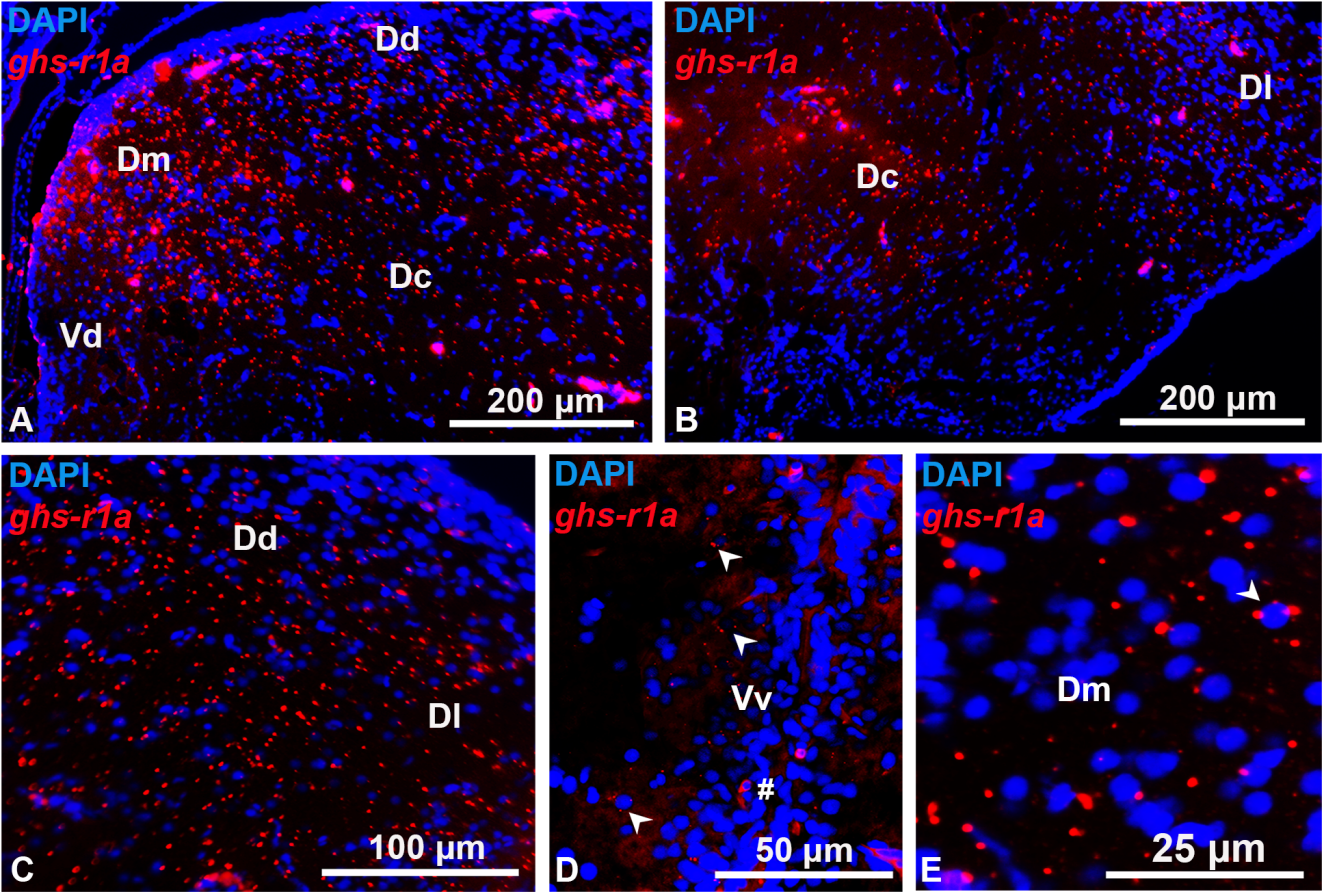
Transversal representative sections of goldfish telencephalon showing *ghs-r1a* positive cells detected by *in situ* hybridization. (A, B) Overview of telencephalon. (C) Lateral portion of the dorsal telencephalon. (D) Ventral portion of the ventral telencephalon (arrowheads indicate riboprobe signaling). (E) Example of telencephalic nucleus surrounded by *ghs-r1a* mRNA riboprobe (arrowhead). Dc, central portion of the dorsal telencephalon; Dd, dorsal portion of the dorsal telencephalon; Dl, lateral portion of the dorsal telencephalon; Dm, medial portion of the dorsal telencephalon; Vd, dorsal portion of the ventral telencephalon; Vv, ventral portion of the ventral telencephalon. Scale bars are indicated in each image.

**Fig 4:**
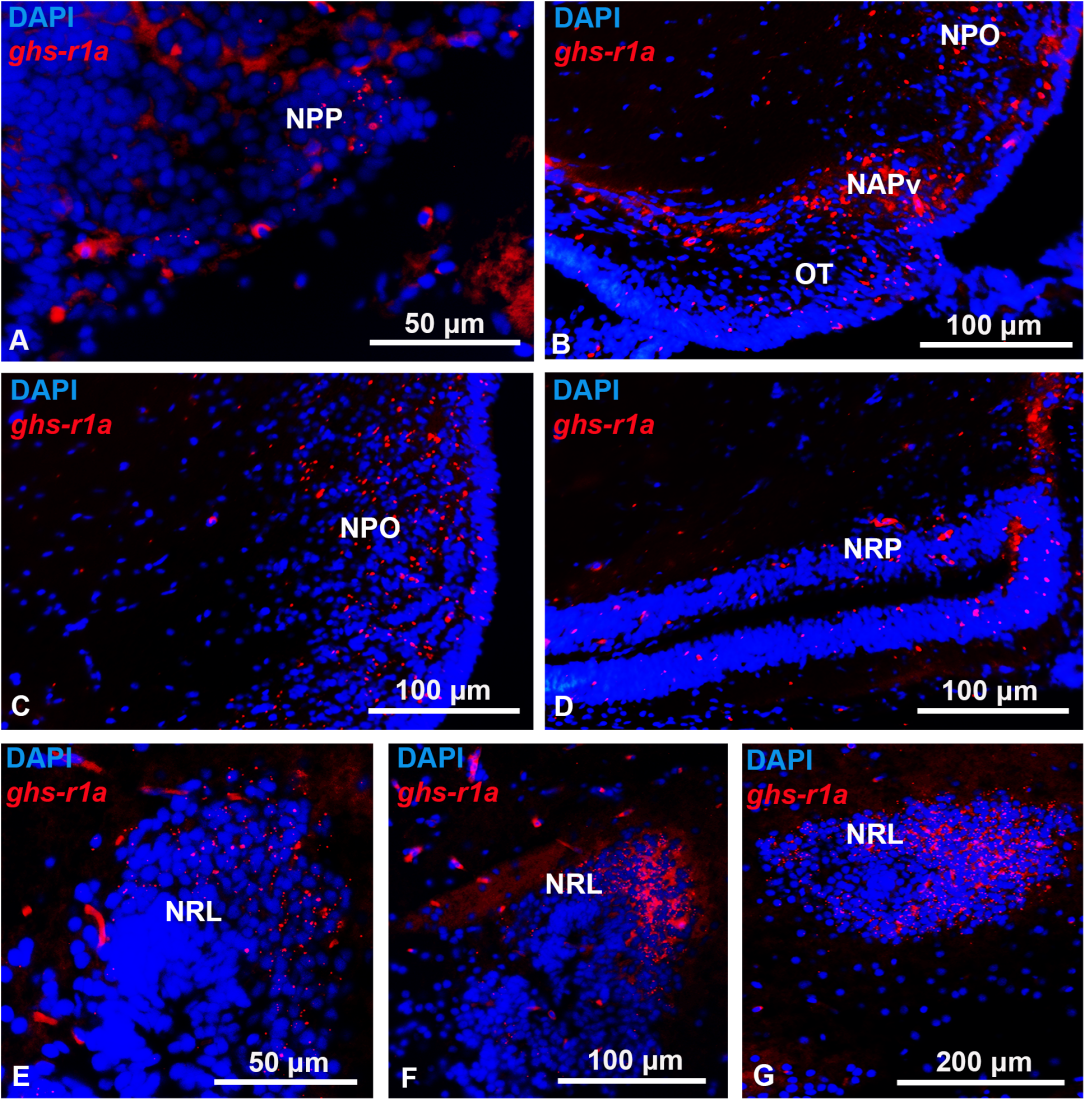
Transversal representative sections of goldfish hypothalamus showing *ghs-r1a* positive cells detected by *in situ* hybridization. (A) Periventricular preoptic nucleus. (B) Preoptic recess. (C) Preoptic nucleus. (D) Posterior recess nucleus. E, F, G. Lateral recess nucleus. NAPv, anterior periventricular nucleus; NPO, preoptic nucleus; NPP, periventricular preoptic nucleus; NRL, lateral recess nucleus; NRP, posterior recess nucleus; OT, optic tract. Scale bars are indicated in each image.

**Fig 5:**
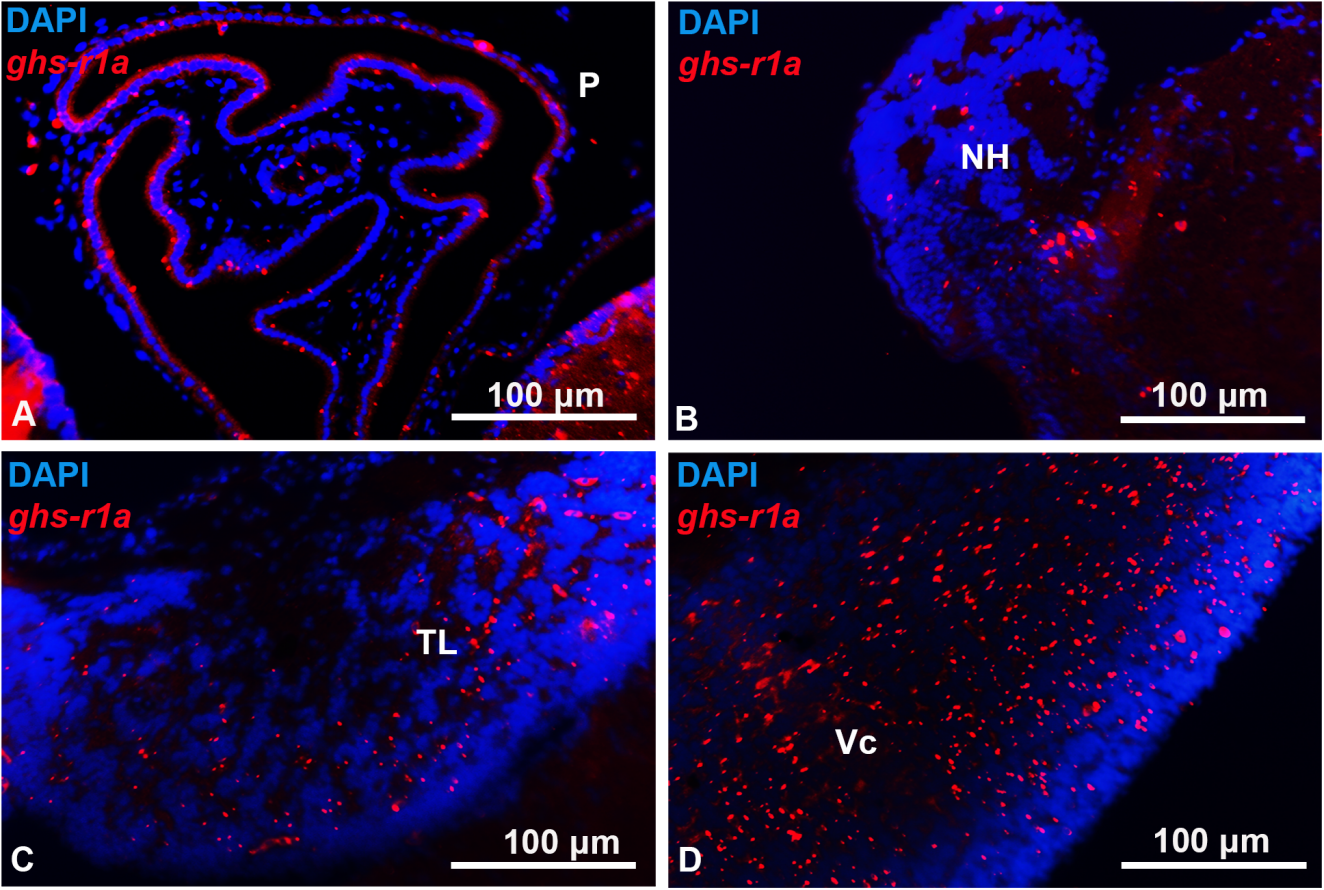
Transversal representative sections of goldfish pineal (A), habenula (B), *torus longitudinalis* (C) and valvula of the cerebellum (D) showing *ghs-r1a* positive cells detected by *in situ* hybridization. NH habenular nucleus; P, pineal; TL, torus longitudinalis; Vc, valvula cerebelli. Scale bars are indicated in each image.

The goldfish brain showed widespread distribution of *ghs-r1a* mRNA (Figs 2–5). Expression of *ghs-r1a* gene within the telencephalon was found in almost all the different areas in the pallial and subpallial regions (Figs 2A, 2B, 2C and 3). In the hypothalamus, *ghs-r1a* expressing cells were detected in the nucleus of the posterior recess (Figs 2D and 4D), the preoptic region, specifically in the periventricular preoptic nucleus (Figs 2B and 4A), and in the nucleus of the lateral recess (Figs 2D, 4E and 4G), with the highest levels of signal observed in the anterior periventricular nucleus (homologous to the mammalian suprachiasmatic nucleus) and in the magnocellular area of the preoptic nucleus (Figs 2B, 2C, 4B and 4C). The *ghs-r1a* expression showed a specific pattern in the nucleus of the lateral recess: it was found only in the lateral area of the most anterior part (Figs 2D and 4E), but it extends to the medial area (Figs 2E and 4F) until *ghs-r1a* mRNA surrounds completely this nucleus in posterior sections (Figs 2F and 4G). There were *ghs-r1a* expressing cells in the pineal gland and in the habenular nuclei (Figs 2D, 5A and 5B). *Ghs-r1a* transcripts were also detected in the *torus longitudinalis* (Figs 2F and 5C) and the highest expression was observed in the valvula of the cerebellum of the metencephalon (Figs 2F and 5D).

**Fig 6:**
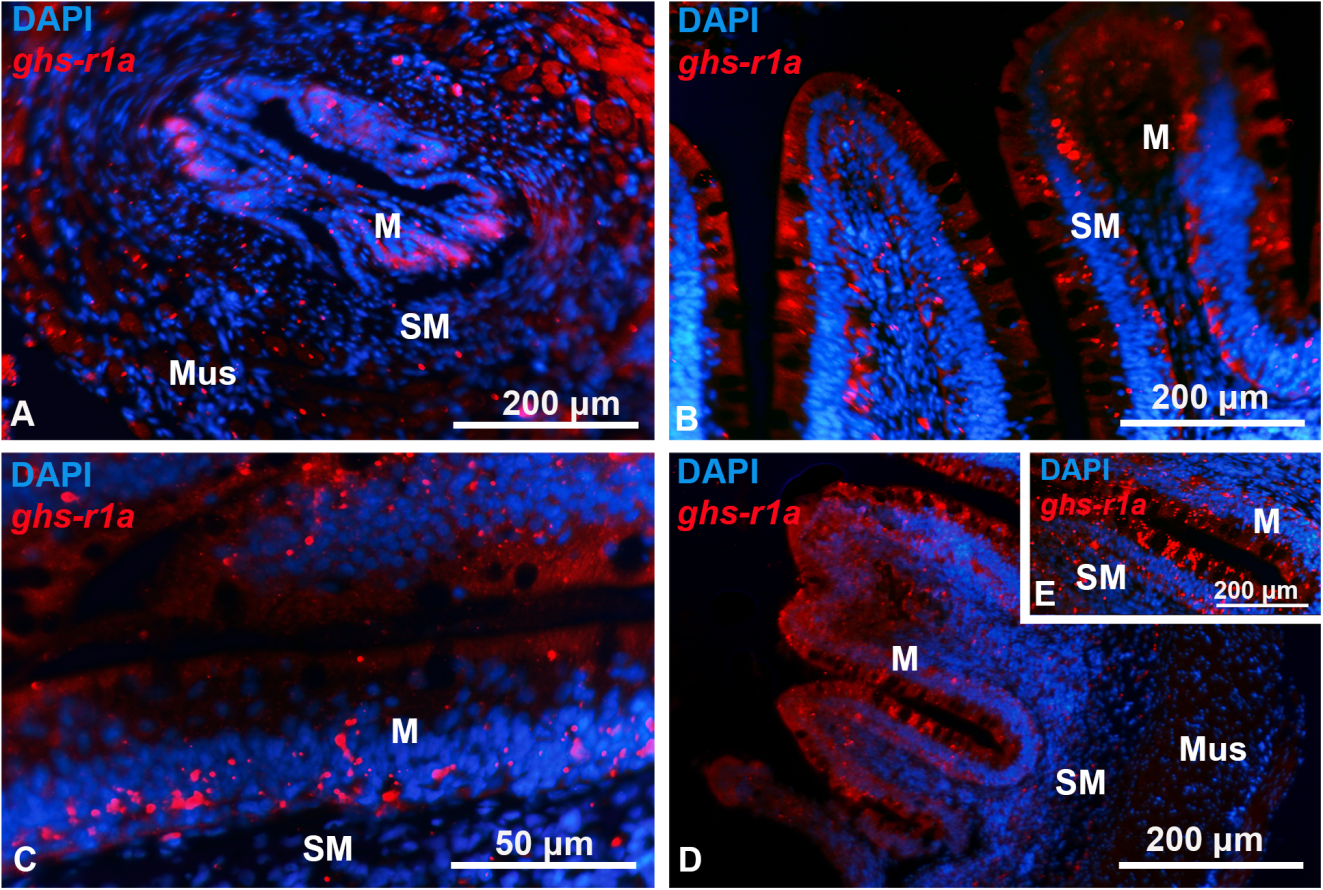
Transversal representative sections of goldfish gastrointestinal tract showing *ghs-r1a* positive cells detected by *in situ* hybridization. (A) Esophagus. (B) Intestinal bulb. (C) j-loop. (D, E) Anterior intestine. M, mucose; Mus, muscular layer; SM, submucose. Scale bars are indicated in each image.

The *ghs-r1a* expression along the gastrointestinal tract of goldfish is shown in Fig 6. A wide distribution was observed in esophagus (Fig 6A), whereas in the intestinal bulb (Fig 6B), j-loop (Fig 6C) and anterior intestine (Fig 6D), *ghs-r1a* expression was observed only in most apical cells of the mucosal epithelium and in the submucosal layer.

### Daily expression of *preproghrelin* and ghs*-r1* ghrelin receptor in goldfish

**Fig 7:**
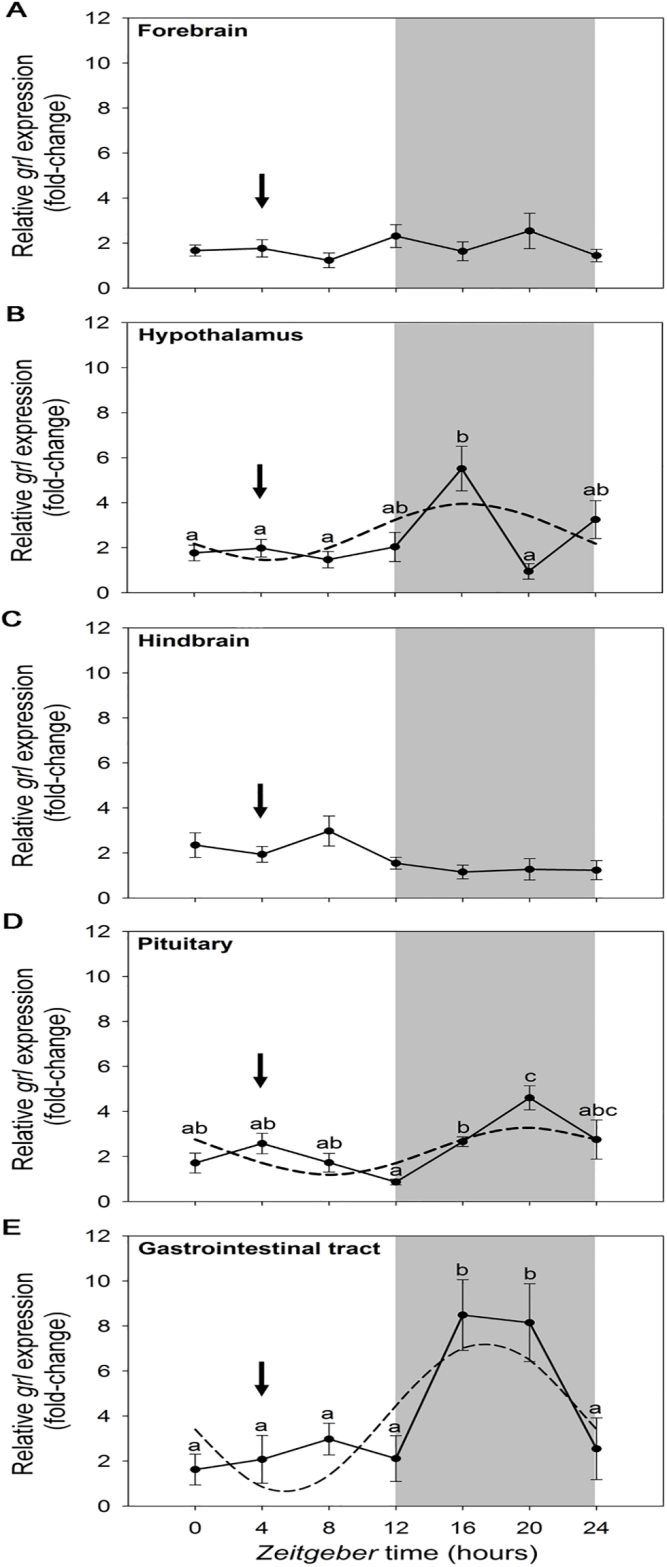
Relative expression of *preproghrelin* in goldfish forebrain (A), hypothalamus (B), hindbrain (C), pituitary (D) and gastrointestinal tract (E) during a 24-h light/dark cycle. Relative mRNA amounts were quantified by RT-qPCR. Data are expressed as mean ± SEM (n = 6/time point). The grey area indicates the dark phase of the daily photocycle, and the arrow indicates the scheduled feeding time (ZT-4). Dashed lines represent the periodic sinusoidal functions determined by the cosinor analysis when a significant rhythm was detected. Different letters indicate significant differences by ANOVA and post-hoc SNK test (p<0.05).

The daily pattern of *preproghrelin* expression in goldfish forebrain, hypothalamus, hindbrain, pituitary and gastrointestinal tract during a 12L:12D photocycle is shown in Fig 7. Statistical analysis (ANOVA and cosinor) showed that *preproghrelin* transcripts displayed significant rhythmic oscillations as a function of the 24-h cycle in hypothalamus (Fig 7B), pituitary (Fig 7D) and gastrointestinal tract (Fig 7E), with acrophases at nighttime. The acrophases in hypothalamus and gastrointestinal tract were advanced around 3–4 h as compared to pituitary (Table 2). The amplitude of *preproghrelin* daily rhythm was 3-fold higher in gastrointestinal tract compared to the hypothalamus and pituitary. No significant differences throughout the 24-h cycle were detected in *preproghrelin* expression in the forebrain and hindbrain.

**Table 2:** Parameters defining the expression rhythms of *preproghrelin* and *ghs-r1* in goldfish.

	Mesor	Amplitude	Acrophase
***Preproghrelin***			
*Hypothalamus*	2.7 ± 0.3	1.2 ± 0.4	16.3 ± 1.3
		*[0*.*1*, *2*.*3]*	
*Pituitary*	2.2 ± 0.2	1.0 ± 0.3	20.0 ± 1.2
		*[0*.*2*, *1*.*8]*	
*Gastrointestinal tract*	3.9 ± 0.5	3.3 ± 0.8	17.4 ± 0.8
		*[1*.*1*, *5*.*5]*	
***ghs-r1***			
*Hypothalamus*	1.5 ± 0.2	0.9 ± 0.2	16.9 ± 0.9
		*[0*.*4*, *1*.*4]*	
*Pituitary*	2.7 ± 0.3	1.4 ± 0.5	19.4 ± 1.2
		*[0*.*1*, *2*.*7]*	

The confidence intervals (99%) of the amplitude values are shown in italics inside the square brackets

**Fig 8:**
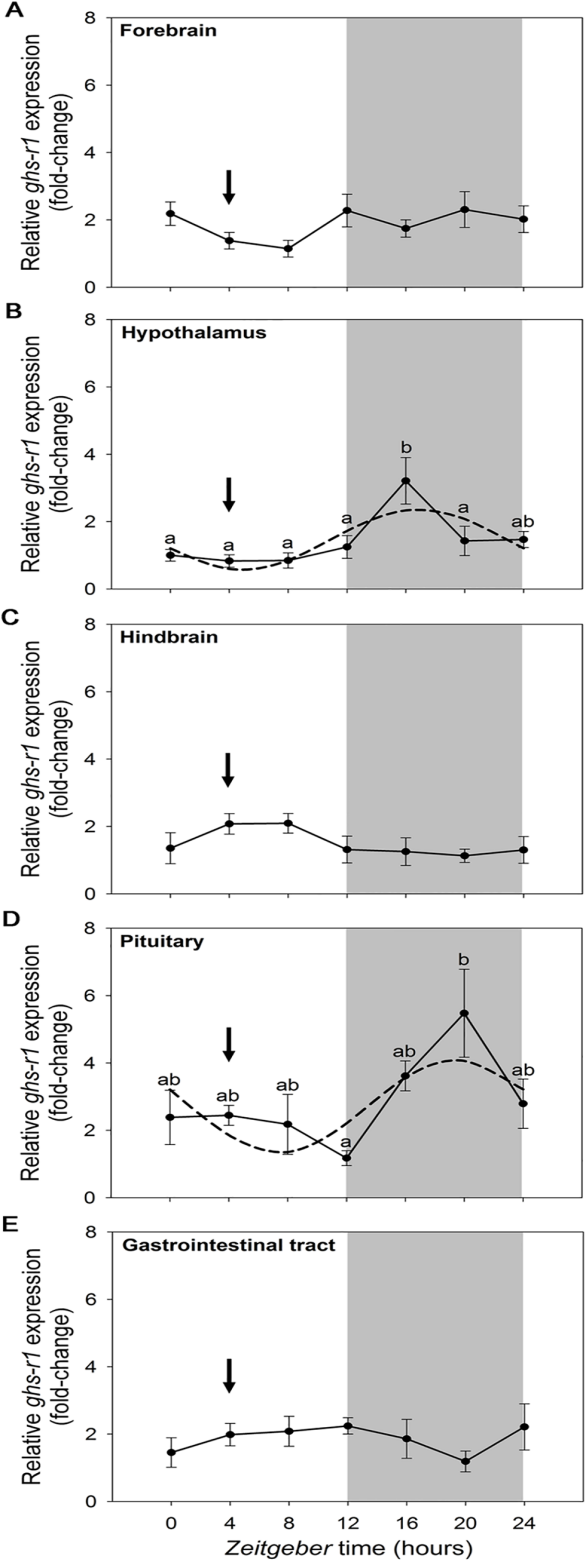
Relative expression of *ghs-r1* in goldfish forebrain (A), hypothalamus (B), hindbrain (C), pituitary (D) and gastrointestinal tract (E) during a 24-h light/dark cycle. Relative mRNA amounts were quantified by RT-qPCR. Data are expressed as mean ± SEM (n = 6/time point). The grey area indicates the dark phase of the daily photocycle, and the arrow indicates the scheduled feeding time (ZT-4). Dashed lines represent the periodic sinusoidal functions determined by the cosinor analysis when a significant rhythm was detected. Different letters indicate significant differences by ANOVA and post-hoc SNK test (p<0.05).

The quantitative analysis of *ghs-r1* daily expression demonstrates the existence of a 24-h rhythmic profile in the hypothalamus and pituitary, with higher abundance of transcripts during the dark phase of the daily photocycle (Fig 8). In both tissues, the acrophase and amplitude of *ghs-r1* daily rhythms were similar to those found for *preproghrelin* (Table 2). Expression of *ghs-r1* was not significantly modified throughout the 24-h cycle in goldfish forebrain, hindbrain and gastrointestinal tract.

## Discussion

The ghrelinergic system is a key regulator of numerous physiological processes, particularly metabolism and reproduction in fish. The brain and gastrointestinal tract are sources of ghrelin, and are major contributors to the endocrine regulation of both, metabolism and reproduction. However, two critically missing information on ghrelin biology are cellular localization of ghrelin transcripts and ghrelin, and the circadian profile of ghrelin in fish tissues. The novel results of this research address both of these paucities in fish ghrelin literature. This study shows for the first time the detailed neuroanatomical distribution of ghrelin and *ghs-r1* in a non-mammal, and the existence of a daily rhythmic expression of *preproghrelin* and *ghs-r1* in the hypothalamus, pituitary, and gastrointestinal tract of goldfish.

Several studies using RT-PCR reported a wide expression of *preproghrelin* mRNA in the brain of fish [5–7] and mammals [3], but its anatomical distribution in discrete encephalic areas remains to be described. Present data support such a broad expression of *preproghrelin* in the goldfish brain by RT-qPCR analyses, but it was undetectable by *in situ* hybridization and immunohistochemical analysis. A possible explanation for these discrepancies might lie on the different age and body weight of fish used for both studies. Thus, it is possible that ghrelin transcript levels in small and immature fish are considerably lower than in big and mature fish, and so be undetected by less sensitive techniques such as ISH and IHC. Furthermore, this is in contrast to a recent study using imaging techniques in which *preproghrelin* was detected in goldfish hypothalamus [13] by IHC. Again, the reproductive stage and metabolic status of fish used in both experiments were significantly different (1.5–2.5 g *vs* 10–20 g) and so might be influencing in the different results observed in one study and the other. This may indicate that detecting ghrelin transcripts in goldfish brain using imaging techniques requires delicate refinement of the techniques used, including higher concentrations of probes/antibodies, longer incubation times, etc.

In the gastrointestinal tract, *preproghrelin* mRNA expression and ghrelin are found by *in situ* hybridization and immunohistochemistry, respectively, with a similar intense signal in all the studied sections. The strong signal detected for *preproghrelin* mRNA expression and ghrelin-like immunoreactivity in proximal sections of the gastrointestinal tract is in accordance with previous studies on the enteric location of this hormone in other vertebrates, where ghrelin was predominantly detected in the anterior part of the gastrointestinal tract. In this respect, ghrelin immunoreactivity decreased from the small to the large intestine was observed in rodents [9], and several studies have reported the stomach as the portion of the intestine with more ghrelin-immunoreactive cells in mammals [1,9,11,12]. Also, in chickens, ghrelin immunoreactive cells were found in the proventriculus and in the small intestine [44]. Finally, in fish, ghrelin immunostaining was predominantly found in the stomach of rainbow trout [16] and sea bass [14], and in the proximal intestine of goldfish [14]. Then, the location of this peptide in the gastrointestinal tract is highly conserved through phylogeny. Furthermore, results from our study show that ghrelin-like immunoreactivity within the gastrointestinal sections is most abundant in the mucosa, which is consistent with previous observations in both mammals [9–12] and fish [14–16]. This observation, together with the fact that *preproghrelin* mRNA was found in the same locations than the peptide, support the mucosal cells as the primary synthesizing site of ghrelin in goldfish.

A second integral component in the ghrelinergic system is its receptor. Our results show that *ghs-r1a* gene is widely expressed in the goldfish brain, from telencephalon to the cerebellum. This is consistent with a previous study describing the expression of the same gene in goldfish brain areas by RT-q PCR, in which expression of *ghs-r1a1* and *ghs-r1a2* was predominantly observed in telencephalon, diencephalon and vagal lobe [23]. In addition, rich expression of *ghs-r1a* is detected in the present study in specific hypothalamic nucleus, such as the lateral recess nucleus, in agreement with the well known orexigenic role of the ghrelinergic system. An interesting observation derived from the present study is that encephalic areas where *ghs-r1a* expression is predominant are known to contain cells that also express other appetite-regulating hormones. For instance, all hypothalamic nuclei expressing *ghs-*r1a, and even some extrahypothalamic locations where *ghs-r1* was found, including the valvula cerebelli, the habenula, the pineal gland and the torus longitudinalis, all have been previously related with the orexinergic system in zebrafish [45,46]. Similarly, we report here an important expression of *ghs-r1a* in the periventricular preoptic nucleus, the anterior part of the periventricular nucleus, the preoptic nucleus and the nucleus of the lateral recess, areas that take part of the NPY system in the European seabass [47] and goldfish [48]. Indeed, a colocalization of the ghrelin receptor and NPY in the hypothalamic arcuate nucleus of the rat, a key nucleus involved in food intake regulation, has been previously reported [28]. This co-expression of both ghrelin receptor and appetite-regulating neuropeptides in feeding-related specific brain areas, as well as the reported physiological interactions among the ghrelinergic system and other orexigenic agents [49–51], support the cross-talking between ghrelin and other orexigenic agents in the feeding regulation.

In the gastrointestinal tract, *ghs-r1a* gene was found mainly in the mucosal epithelium of all the areas studied, matching the expression of *preproghrelin* gene. However, interestingly, the expression of the receptor was highest in the most apical cells of mucosal folds, while highest levels of the prepropeptide were observed in the most basal cells of this epithelium. Ghrelin receptor immunoreactivity in the gastrointestinal tract has only been reported in zebrafish by Olsson and co-workers [15], who described the presence of the receptor in numerous endocrine cells of the mucosa and in the muscle layers along the entire intestine. This important presence of ghrelin receptors in the muscle layers of the zebrafish gut was suggested to be related with motility functions of ghrelin in this teleost [15]. In the present study, *ghs-r1a* expressing cells in the muscle layer were only observed in the esophagus, but not in the intestinal bulb, j-loop and anterior intestine. In fact, it is to note that ghrelin was ineffective on gut motility in rainbow trout and goldfish [52], which is consistent with present results on the absence of ghrelin receptors in the muscle layers of goldfish gastrointestinal tract, but in contrast with results on zebrafish [15]. Together, here we show the brain-gut mapping of ghrelin and its receptor, two main components of the ghrelinergic system, in goldfish. GOAT, the third peptide in this hormonal system, is yet to be identified in goldfish and future studies warrant its localization.

Previous research [53] shows ghrelin is a multifunctional peptide in fish, and our current results show extensive, yet cell specific localization of ghrelin and its receptor in goldfish tissues. Is there a daily pattern of expression for ghrelin and GHS-R in goldfish? In fact, recent studies in this teleost show that some food intake regulatory hormones, such as NPY [29] and leptin [30], display daily oscillations in response to the 24-h light/dark cycle, suggesting a relationship between the orexigenic system and circadian organization. Such a relationship between ghrelin and the circadian system has been pointed out in mammals, where the stomach ghrelin-secreting cells were described to contain the machinery that constitutes a food entrainable oscillator [54]. However, no studies to date have analyzed daily changes in expression of *preproghelin* and its receptors in any vertebrate group. Our results demonstrate daily rhythms of *preproghrelin* and *ghs-r1* expression in hypothalamus and pituitary of goldfish maintained under scheduled photoperiod and feeding regime. Considering the key role played by the hypothalamus in the functional organization of the circadian system [55], the existence of daily rhythms in the hypothalamic ghrelinergic system supports such interplay between ghrelin and the circadian system. Moreover, it is important to note that a high expression of *ghs-r1a* was detected in specific hypothalamic nucleus related with the circadian organization, such as the anterior periventricular nucleus, which is homologous to the mammalian suprachiasmatic nucleus, the master clock regulating circadian functions in these vertebrates [56]. In the periphery, the daily expression pattern found for *preproghrelin*, but not for *ghs-r1* in the gastrointestinal tract, might suggest that the daily regulation of ghrelin-related genes in peripheral organs is exerted only on bioactive peptide synthesis, without effect on its receptors. It is also interesting that the amplitude of the *preproghrelin* expression rhythm is nearly 3-fold higher in the gastrointestinal tract compared to hypothalamus and pituitary. This strength in rhythmicity reinforces the proposal that ghrelin might be an important output of the intestinal oscillator.

Present results revealed a major finding regarding the nocturnal acrophase of the *preproghrelin* and *ghs-r1* expression rhythms in both central and peripheral locations. The biological significance of *preproghrelin* and *ghs-r1* transcripts peaking during the night remains to be elucidated, although it could be related with any of the wide variety of physiological functions that ghrelin is known to exert apart from feeding regulation in non-mammalian vertebrates, i.e. growth, reproduction, immunity [53]. Interestingly, two main observations are to note for a possible crosstalking between the ghrelinergic and the circadian systems. On one hand, the 24-h ghrelinergic expression profile overlaps the one described for melatonin, a key component of the circadian system in vertebrates [57]. On the other, the rhythms are also in phase with the daily rhythms of the negative loop clock genes of the clock molecular machinery (*gper1a*, *gper1b*, *gper3* and *gcry3* in the hypothalamus [58,59] and gut [60,61] of goldfish), and are in antiphase with the genes of the positive loop (*clock1a* and *bmal1* in rainbow trout [62] and senegale sole [63]). Aditionally, ghrelin was found to induce the expression of some *per* and *cry* genes, but not *bmal1a*, in goldfish central and peripheral tissues [37]. The possible connection between the ghrelinergic system with negative elements of the circadian molecular machinery, such as *per*, is also supported by the similar anatomical distribution reported in present study for *ghs-r1a* gene in brain, and the one previously reported for *gper1b* gene in goldfish maintained under the same environmental conditions [59]. All these parallelisms clearly suggest a connection between the ghrelinergic system and clock genes expression, although further studies are required to demonstrate the specific implication of ghrelin in this function.

In summary, present results demonstrate that *preproghrelin*, ghrelin and ghrelin receptor *ghs-r1a* are widely expressed in the goldfish brain and gastrointestinal tract, and show for the first time a rhythmical pattern of expression in hypothalamus, pituitary and gastrointestinal tract of goldfish. These rhythmic patterns indicate an important connection between the ghrelinergic system and the circadian system of teleosts, and suggest that ghrelin might be acting alternatively as an input/output of the food entrainable oscillator.

## Supporting Information

S1 Fig Specificity of *preproghrelin* and *ghs-r1a* mRNA riboprobes and the antibody anti-human ghrelin. A, B. Anterior intestine showing *preproghrelin* antisense riboprobes signaling (arrowheads) surrounding the nucleus. C. Anterior intestine showing *preproghrelin* sense riboprobes staining. D, E. Telencephalon showing *ghs-r1a* antisense riboprobes signaling (arrowheads) staining. F. Telencephalon showing *ghs-r1a* sense riboprobes staining. G. Antibody anti- human ghrelin cytoplasmic signal (arrowheads) in the anterior intestine. H. Control anterior intestine without the primary antibody, incubated only with the secondary one. #: Blood cells with unspecific staining.
(PDF)
