# Supplementary material for: In Situ Localization and Rhythmic Expression of Ghrelin and ghs-r1 Ghrelin Receptor in the Brain and Gastrointestinal Tract of Goldfish (Carassius auratus)
Source: PLoS One. 2015 Oct 27;10(10):e0141043. doi: 10.1371/journal.pone.0141043 (PMC4624692; doi:10.1371/journal.pone.0141043)
Supplement: S1 Fig — A, B. Anterior intestine showing preproghrelin antisense riboprobes signaling (arrowheads) surrounding the nucleus. C. Anterior intestine showing preproghrelin sense riboprobes staining. D, E. Telencephalon showing ghs-r1a antisense riboprobes signaling (arrowheads) staining. F. Telencephalon showing ghs-r1a sense riboprobes staining. G. Antibody anti- human ghrelin cytoplasmic signal (arrowheads) in the anterior intestine. H. Control anterior intestine without the primary antibody, incubated only with the secondary one. #: Blood cells with unspecific staining. (PDF) [file pone.0141043.s001.pdf]

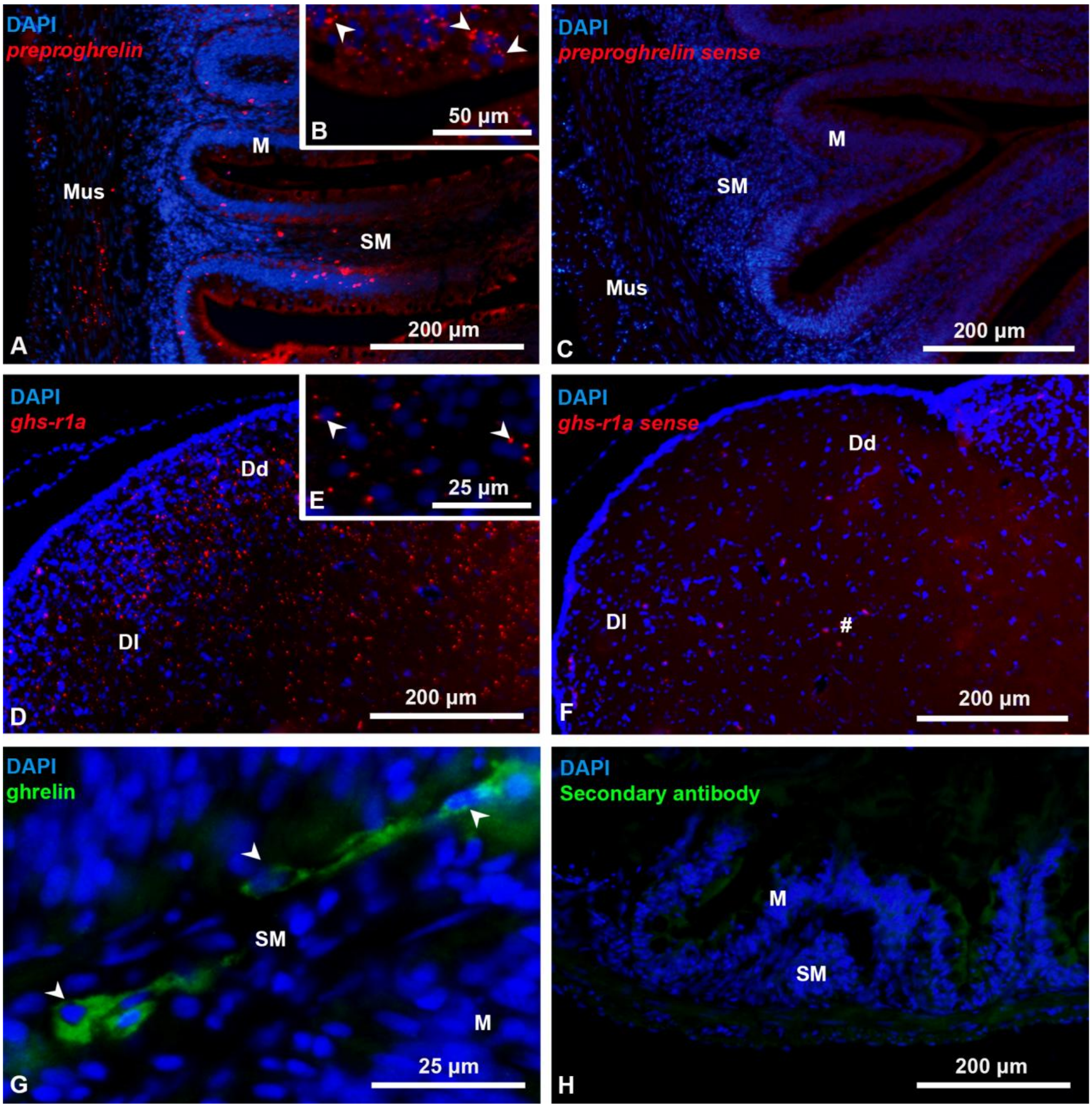

S1. Fig. Specificity of *preproghrelin* and *ghs-r1a* mRNA riboprobes and the antibody anti-human ghrelin. A, B. Anterior intestine showing *preproghrelin* antisense riboprobes signaling (arrowheads) surrounding the nucleus. C. Anterior intestine showing *preproghrelin* sense riboprobes staining. D, E. Telencephalon showing *ghs-r1a* antisense riboprobes signaling (arrowheads) staining. F. Telencephalon showing *ghs-r1a* sense riboprobes staining. G. Antibody anti- human ghrelin cytoplasmic signal (arrowheads) in the anterior intestine. H. Control anterior intestine without the primary antibody, incubated only with the secondary one. #: Blood cells with unspecific staining.
